# Training in endocrine surgery

**DOI:** 10.1007/s00423-019-01828-4

**Published:** 2019-11-07

**Authors:** Oliver Gimm, Marcin Barczyński, Radu Mihai, Marco Raffaelli

**Affiliations:** 1 Department of Surgery and Department of Clinical and Experimental Medicine (IKE), Linköping University, 58183 Linköping, Sweden; 2 Department of Endocrine Surgery, Third Chair of Surgery, Jagiellonian University Medical College, 37 Prądnicka Street, 31-202 Kraków, Poland; 3 Department of Endocrine Surgery, Churchill Cancer Centre, Oxford University Hospital NHS Foundation Trust, Oxford, OX3 7DU United Kingdom; 4 U.O. Chirurgia Endocrina e Metabolica, Fondazione Policlinico Universitario A. Gemelli IRCCS, Rome, Italy; 5 Istituto di Semeiotica Chirurgica, Università Cattolica del Sacro Cuore, Rome, Italy

**Keywords:** Training, Resident, Fellow, Endocrine, Surgery

## Abstract

**Background/purpose:**

In Europe, the Division of Endocrine Surgery (DES) determines the number of operations (thyroid, neck dissection, parathyroids, adrenals, neuroendocrine tumors of the gastro-entero-pancreatic tract (GEP-NETs)) to be required for the European Board of Surgery Qualification in (neck) endocrine surgery. However, it is the national surgical boards that determine how surgical training is delivered in their respective countries. There is a lack of knowledge on the current situation concerning the training of surgical residents and fellows with regard to (neck) endocrine surgery in Europe.

**Methods:**

A survey was sent out to all 28 current national delegates of the DES. One questionnaire was addressing the training of surgical residents while the other was addressing the training of fellows in endocrine surgery. Particular focus was put on the numbers of operations considered appropriate.

**Results:**

For most of the operations, the overall number as defined by national surgical boards matched quite well the views of the national delegates even though differences exist between countries. In addition, the current numbers required for the EBSQ exam are well within this range for thyroid and parathyroid procedures but below for neck dissections as well as operations on the adrenals and GEP-NETs.

**Conclusions:**

Training in endocrine surgery should be performed in units that perform a minimum of 100 thyroid, 50 parathyroid, 15 adrenal, and/or 10 GEP-NET operations yearly. Fellows should be expected to have been the performing surgeon of a minimum of 50 thyroid operations, 10 (central or lateral) lymph node dissections, 15 parathyroid, 5 adrenal, and 5 GEP-NET operations.

## Introduction

Endocrine surgery is a subspecialty focusing on the surgical management of various diseases of the endocrine glands, including the thyroid gland, the parathyroid glands, the adrenal glands, and neuroendocrine tumors (NET) of the gastrointestinal tract and pancreas. These conditions are selectively managed by general surgeons, upper and lower gastrointestinal surgeons, surgical oncologists, otolaryngologists, head and neck surgeons, and urologists depending on their surgical training backgrounds, professional interests, and referral patterns reflecting the local healthcare environment.

Training of surgical trainees has always been challenging and endocrine surgery is no exception. During residency, surgical trainees are confronted with a variety of different surgical procedures in order to become a surgical specialist. Following residency, many surgical specialists decide to undertake a fellowship period in order to develop a subspecialty expertise, for example in endocrine surgery. While surgical residents are required to learn the basics of many surgical procedures, fellows are required to acquire a much more profound knowledge on (almost) every surgical aspect of their chosen subspecialty. Logically, fellows in endocrine surgery have to be confronted with many more cases than surgical residents.

In order to ensure that the training has reached the required level of knowledge and skills, standardized examinations are mandatory. In Europe, the Division of Endocrine Surgery (DES) is responsible for organizing such standardized examinations in endocrine surgery on behalf of the Section of Surgery of the European Union of Medical Specialist (UEMS). Successful candidates get a European Board of Surgery Qualification (EBSQ) certificate. The DES issues EBSQ certificates in neck endocrine surgery (i.e., thyroid gland and parathyroid gland surgery and knowledge of the underlying diseases) or endocrine surgery (including even the adrenal glands and NETs of the gastro-entero-pancreatic (GEP) tract). As of May 16, 2019, 108 surgeons had successfully passed the EBSQ examination in endocrine surgery and 54 surgeons/otolaryngology doctors had successfully passed the counterpart in neck endocrine surgery. Successful candidates are considered fellows of the DES and entitled to bear the title Fellow of the European Board of Surgery (FEBS—endocrine surgery or FEBS—neck endocrine surgery).

The DES has an executive committee that is elected by national delegates. Currently, the DES has national delegates from 28 countries. These delegates are also the national delegates of the European Society of Endocrine Surgeons (ESES). The minimum number of operations required to be eligible for the EBSQ exam in (neck) endocrine surgery was determined by the national delegates before the first examination took place in 2003 (Table 1). Though the DES determines the number of operations to be required for the exam in (neck) endocrine surgery, it is the European national surgical boards that determine how surgical training is delivered in their respective countries.

**Table 1:** Currently (May 2019) recommended minimal operative experience to be eligible for the European Board of Surgery Qualification in endocrine and neck endocrine surgery as defined by the Division of Endocrine Surgery, UEMS

	Performed	Assisted
Operations		
Thyroid resections^§^	50	50
Recurrent thyroid operation^§^		5
Central compartmental lymph node clearance^§^	2	15
Lateral compartment lymph node clearance^§^	2	10
Parathyroidectomy in HPT^§^	15*	20*
Adrenalectomy^#^	2	10
Resection for NET of the GI tract^#^	2	5

*At least 10 bilateral explorations demanded

^§^Required for both the exam in endocrine and neck endocrine surgery

^#^Required only for the exam in endocrine surgery

So far, however, the requirements set out by the European national surgical boards with regard to endocrine surgery have not been investigated. In May 2019, the ESES organized the 8th biennial conference with the topic “Volume outcomes and quality in endocrine surgery.” One working group was tasked to evaluate the current situation on the training of general surgery/otolaryngology residents in endocrine surgery and of endocrine surgery fellows. The members of the working group are the authors of this manuscript.

## Material and methods

During October and November 2018, we performed a survey by sending out two questionnaires to all 28 current national delegates of the DES/ESES. One questionnaire was addressing the training of surgical residents while the other was addressing the training of fellows in endocrine surgery. The questionnaires were also sent to a few non-European endocrine surgeons. Their responses are briefly addressed in the discussion.

The national delegates were asked regarding the current minimum numbers of operations deemed necessary for the completion of training in their respective countries and concerning the numbers of operations they themselves considered to be appropriate for being competent with regard to independent practice in surgery of the thyroid gland including neck dissections, the parathyroid glands, the adrenal glands, and NETs of the gastro-entero-pancreatic tract. In general, we distinguished between operations participated in as the performing (main) surgeon and as the assisting surgeon. Additional questions were asked about the structure/content of the existing postgraduate examinations.

In preparing this manuscript, the authors also searched the PubMed database by using various combinations of the following search terms: surgery, training, resident/residency, fellow(ship), thyroid(ectomy), parathyroid(ectomy), adrenal(ectomy), neuroendocrine, gastric, intestine, pancreas, and outcomes.

## Results

### Personal views of the national delegates and existing examinations

#### Surgical residents

A total of 24 national delegates (86% of 28) of the DES responded to the questionnaire on the training of surgical residents. Only half of the responders considered that 20 or more endocrine surgical procedures operations should be mandatory (Table 2). At the low end of this spectrum, one respondent indicated that 5 or less performed procedures would be acceptable.

**Table 2:** Recommended minimal operative experience of endocrine surgical operations for surgical residents and fellows in endocrine surgery as defined by the national delegates of the Division of Endocrine Surgery/European Society of Endocrine Surgeons

Number of minimal operations recommended	Number of national delegates agreeing on this number
Surgical residents	
≥ 50	4
20–49	10
10–19	3
5–9	2
< 5	1
Fellows in endocrine surgery	
≥ 200	3
100–199	3
50–99	3
< 50	1

A final written exam followed by an oral exam at the end of surgical residency is mandatory in 10 European countries. An oral exam only is mandatory in another 10 European countries whereas no mandatory exam exists in four European countries. A practical exam (e.g., OSCE) is organized in 12 European countries and a three-stage exam (written, oral, and practical) is mandatory in six European countries.

#### Fellows in endocrine surgery

Sixteen national delegates (57% of 28) replied to the questionnaire on the training of fellows in endocrine surgery. Only three respondents considered that 200 or more endocrine surgical procedures should be performed during fellowship training in endocrine surgery, whereas 3 respondents considered 100–199 performed procedures to be sufficient. Three respondents agreed that 50–99 performed procedures would be a minimum (Table 2). Only 1 respondent indicated that less than 50 procedures performed would be acceptable, whereas 6 respondents skipped to answer this question. A written exam followed by an oral exam at the end of the fellowship in endocrine surgery is mandatory in three European countries, and an oral exam only is mandatory in one European country, whereas no obligatory exam exists in 12 European countries. A practical exam (e.g., OSCE) is organized in only one European country and it is a part of a three-stage exam (written, oral, and practical).

### Training in thyroid gland surgery and neck dissection

#### Survey results

Programs for surgical residents contained thyroid procedures in the majority (*n* = 22) but not all European countries (*n* = 2). The minimum number of total/near-total thyroidectomies to be performed/assisted during residency varied between 0 and 40 (median 0.5)/0 and 50 (median 0) and these values were far below the values expected to be sufficient from the national delegates’ perspective: 0–50 (median 10) and 0–50 (median 20), respectively. The minimum number of hemithyroidectomies (or thyroid resections) to be performed/assisted during residency ranged from 0 to 50 (median 0)/0 to 50 (median 0) and these values were also below the values expected to be required from the national delegates’ perspective: 0–50 (median 15) and 0–50 (median 20), respectively. The minimum number of central lymph nodes dissections to be performed/assisted varied from 0 to 20 (median 0) and 0 to 20 (median 0), respectively. The national delegates’ expectations were slightly higher in this regard: 0–20 (median 5) and 0–50 (median 10), respectively. The minimum number of lateral lymph nodes dissections to be performed/assisted during surgical residency ranged from 0 to 10 (median 0) and 0 to 20 (median 0), respectively. The national delegates’ expectations were relatively consistent with the current minimum requirement for performed operations with 0–10 (median 2) but much higher for assisted lateral neck dissections with 0–20 (median 5).

**Fig. 1:**
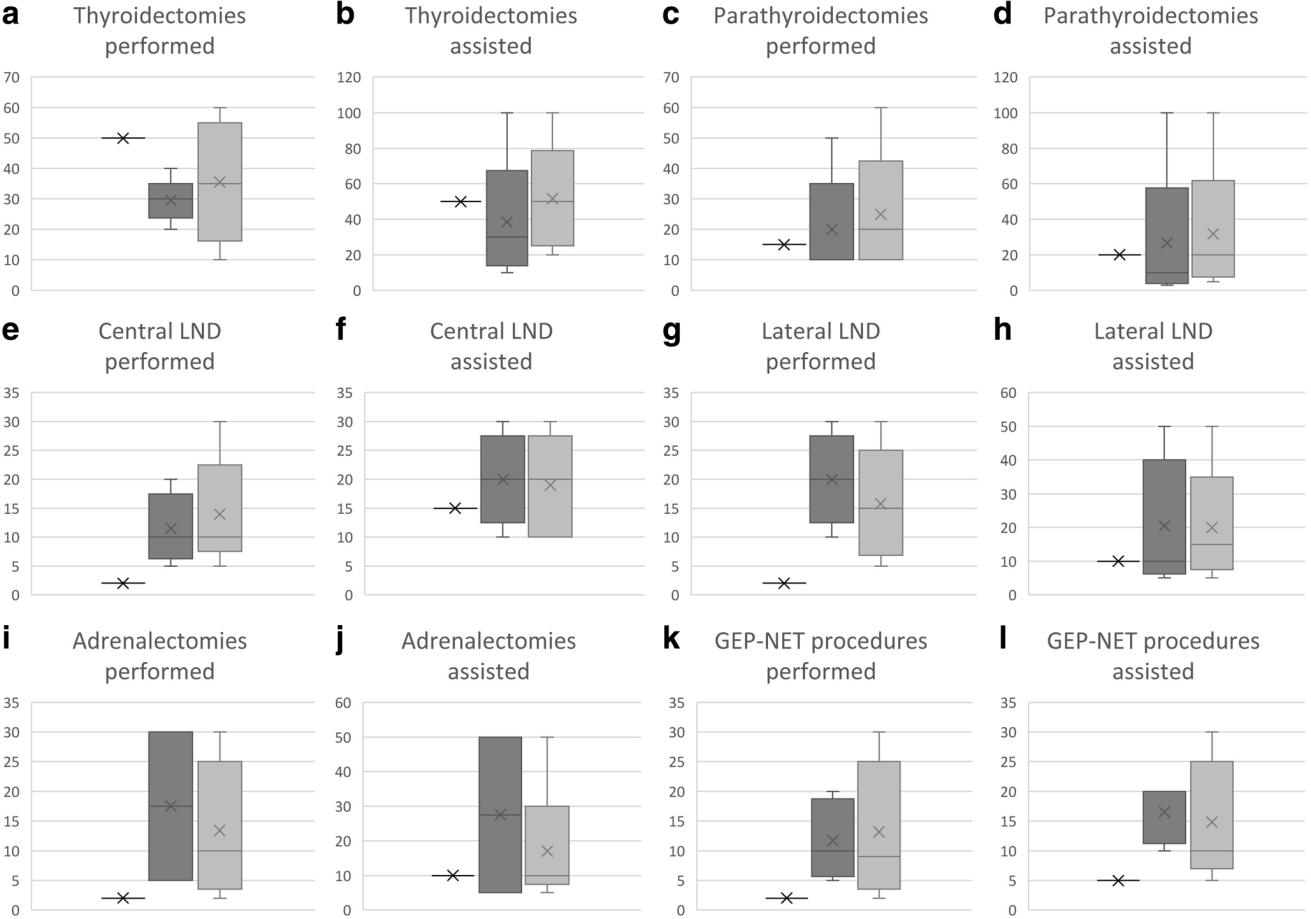
Number of operations considered appropriate for fellows in endocrine surgery regarding thyroidectomies (**a**, **b**), parathyroidectomies (**c**, **d**), central (**e**, **f**) and lateral (**g**, **h**) lymph node dissections (LND), adrenalectomies (**i**, **j**) and various surgical procedures concerning gastro-entero-pancreatic neuroendocrine tumors (GEP-NET) (**k**, **l**). Horizontal black line, current (May 2019) requirements as defined by the Division of Endocrine Surgery. Dark gray boxplot, current requirements as defined by European national surgical boards. Bright gray boxplot, recommended numbers by European national delegates

With regard to fellows (Fig. 1), our survey revealed that the programs for fellowships in endocrine surgery contained thyroid procedures in all European countries which offer some form of fellowship in this area. The minimum numbers of total/near-total thyroidectomies to be performed/assisted during fellowship varied from 20 to 30 (median 30)/0 to 100 (median 30) and these values were somewhat below the values expected to be optimal from the national delegates’ perspective with 10–60 (median 30) and 20–100 (median 50), respectively. The minimum numbers of hemithyroidectomies (or thyroid resections) to be performed/assisted during fellowship varied from 20 to 40 (median 30)/10 to 50 (median 25) and theses values were also below the values expected to be optimal from the national delegates’ perspective with 20–60 (median 40) and 20–100 (median 50), respectively. The minimum numbers of central lymph node dissections to be performed/assisted varied from 5 to 20 (median 10)/10 to 30 (median 20), and the national delegates’ expectations were very similar in this regard with 5–30 (median 10) and 10–30 (median 20), respectively. The minimum numbers of lateral lymph nodes dissections to be performed/assisted during fellowship varied from 10 to 30 (median 20)/5 to 50 (median 10), and national delegates’ expectations were very similar for both performed operations with 5–30 (median 15) and assisted lateral neck dissections with 5–50 (median 15).

#### Literature regarding training in thyroid surgery and neck dissection

The current training of general surgical residents with regard to thyroid and other types of endocrine operations is highly variable, which may contribute to increased complication rates and number of reoperations [1]. The mean minimum number of thyroid operations to be done during surgical residency as shown by this survey was 5 performed and 13 assisted procedures, and these values were two-fold lower than what was recommended by the national delegates on average. This issue raises the question if a general surgeon with no special interest in thyroid surgery has received enough exposure during residency to be able to perform a total thyroidectomy safely.

Total thyroidectomy is an operation that always engenders controversy related to injury of the recurrent laryngeal nerves and the parathyroid glands. It has been shown that surgeons who have completed a well-designed training program and who have become proficient in total thyroidectomy as trainees will remain proficient despite practicing in a provincial center [2]. Achieving a low morbidity rate demands meticulous attention to operative technique and anatomical details [2].

Interesting insight into this issue was recently provided by Phitayacorn et al. who published an expert consensus of general surgery residents’ proficiency concerning common endocrine operations. In this study, members of the American Association of Endocrine Surgeons (AAES) were surveyed about their opinions on resident proficiency with regard to common endocrine operations [3]. A total of 92% of the respondents operated with residents. On average, they believed that most of the steps of a total thyroidectomy for benign disease could be performed by a postgraduate year 4 surgery resident. Specific steps that were considered to require more training included decisions to divide the strap muscles or leaving a drain. Approximately 66% of the respondents felt that a postgraduate final-year surgery resident could independently perform a total thyroidectomy for benign disease but only 45% felt similarly for malignant thyroid disease. The annual endocrine volume of the respondents did not correlate with the beliefs concerning the residents’ autonomy. There was general agreement that a postgraduate final-year resident may not be proficient in advanced endocrine operations. Nevertheless, it was also felt that opportunities exist to improve the training for graduates that anticipate performing endocrine operations routinely [3, 4]. For more data on pertinent training issues on thyroid surgery see Table 3.

**Table 3:** Summary of relevant publications on training in endocrine surgery

Reference	Publication date	Main outcomes
Harness et al. [5]	1995	During 8 academic years (1986–1994), the average number of thyroidectomies performed by resident graduating of US general surgery programs ranged from 10.3 to 12.6 (maximum from 52 to 102), with the most common number of thyroidectomies performed ranged from 7 to 10 per graduating resident. For parathyroidectomy, the average ranged from 4.1 to 5.1, the maximum ranged from 25 to 60, and the most common number performed was 2.
Prinz [6]	1996	During the academic years 1994–1995, the number of endocrine procedures performed per resident of US general surgery programs increased. In particular, thyroidectomy 13.5 ± 5.8 (range 3–35), parathyroidectomy 6.1 ± 3.4 (range 1–48). In residency programs with one or more endocrine surgeon(s) in the teaching faculty, the number of thyroidectomies (14.5 ± 5.4 vs 12.5 ± 6.1) and parathyroidectomies (7.3 ± 3.7 vs 4.9 ± 2.5) was significantly higher.
Parsa et al. [7]	2000	The overall operative volume of US general surgery resident increased from 1991 to 1997. In particular, there was a 19.2% increase in the average number of thyroidectomies (14.2 vs 12.1) and a 51.2% increase in the number of parathyroidectomies performed (6.1 vs 4.4).
Manolidis et al. [8]	2001	The results of thyroid surgery performed by residents in training in an otolaryngology—head and neck—surgery program in a metropolitan public hospital, measured by rates of complications, length of hospitalization, and duration of surgery, are similar to those of faculty at a private hospital setting in groups of patients with very similar characteristics.
Sosa et al. [9]	2007	From 2001 to 2006, US graduating general surgery chief residents on average have performed < 30 endocrine procedures (18 thyroidectomies, 8.6 parathyroidectomies, 1.8 adrenalectomies, 0.1 operations for neuroendocrine tumors of the pancreas, 1.5 neck dissections). From 2003 to 2006, the average number of endocrine procedures during US endocrine surgery fellowship was 253 (range 107–445), including 127 thyroidectomies, 90 parathyroidectomies, 15 neck dissections, 15 adrenalectomies, and 3.0 pancreas procedures.
Terris et al. [10]	2007	There was a gradual increase in the mean number of parathyroidectomies performed by US general surgery residents from 6.0 in 1996 to a peak of 9.2 in 2004; this volume has begun to decline in 2005 (to 8.5). During the same timeframe, the mean number of parathyroidectomies performed by OHNS residents rose sharply and steadily from 1.8 in 1996 to 10.9 in 2005.
Le et al. [11]	2008	Between 1995 and 2004, there was a gradual increase in the mean number of endocrine surgical procedures by US general surgery residents (thyroidectomies from 13.2 to 18.2, parathyroidectomies from 5.6 to 9.2, adrenalectomies from 1.2 to 1.7) with the exception of endocrine pancreas resection (from 0.2 to 0.1) and other major endocrine procedures (0.1 to 0.1). US fellowship programs showed significant differences in the number of endocrine operative cases performed at each program ranging from 27 to 732 (thyroidectomies 15 to 500, parathyroidectomies 10 to 500, adrenalectomies 1 to 75, endocrine pancreas 1 to 100, GI endocrine 0 to 40).
Goldfarb et al. [12]	2010	Between 2005 and 2008, at hospitals participating in the National Surgical Quality Improvement Program (NSQIP) of the American College of Surgeons, senior residents assisted in 36.5% of 29,161 endocrine operations (51.7% of 1781 adrenalectomies, 34.9% of 18,279 thyroidectomies and 36.7% of 9101 parathyroidectomies). Junior residents assisted in 30.6% of the total cases (11.3% of adrenalectomies, 31.3% of thyroidectomies, and 32.9% of parathyroidectomies). Fellows assisted in 6.6% of the total cases (18.3% of adrenalectomies, 4.7% of thyroidectomies, and 8.2% of parathyroidectomies). Trainees-assisted operations were associated with longer operative time and shorter hospital stay but no difference in complications rate.
Solorzano et al. [13]	2010	Survey among endocrine surgeons in practice < 7 years and endocrine surgery fellows. Endocrine surgery fellows performed significantly more endocrine surgery cases in residency than the average graduating GS residents (45 vs 18 thyroidectomies, 26 vs 9 parathyroidectomies, 6 vs 2 neck dissections, 6 vs 2 laparoscopic adrenalectomies, 4 vs 0 pancreatic resections). The estimated mean number of performed procedures to be competent was 60 thyroidectomies, 50 parathyroidectomies, 15 laparoscopic adrenalectomy, 12 neck dissections, and 12 endocrine pancreas resections. Fellows graduated with a median (range) of 150 (50–300) thyroid, 80 (35–200) parathyroid, 10 (2–50) neck dissection, 13 (0–60) laparoscopic adrenal, and 3 (0–35) endocrine pancreas. Fellows felt the least prepared in neck dissection and pancreas.
Zarebczan et al. [14]	2010	Between 2004 and 2008, the average endocrine surgery volume of US general surgery and otolaryngology residents increased by approximately 15% (26.4 to 30.9 cases and 57.1 to 67.3, respectively). The growth in case volume was mostly from increases in the number of thyroidectomies performed by US general surgery and otolaryngology residents (17.9 to 21.8 and 46.5 to 54.4, respectively). Overall, there was an increase also in the number of parathyroidectomies (8.5 vs 9.1 and 10.6 vs 12.9, respectively). Most general surgery residents performed thyroidectomies and parathyroidectomies earlier in their training as surgeon juniors. Conversely, otolaryngology residents are performing most thyroidectomies and parathyroidectomies as chief residents.
Monteiro et al. [15]	2013	More thyroid/parathyroid operations are performed with residents in general surgery than ENT; junior residents in general surgery perform a large percentage of these cases (about 40%), indicating early exposure to endocrine surgery and balanced experience between resident levels with minimal effect of fellows. Although junior residents receive exposure in ENT, a greater proportion is performed by fellows.
Reinisch et al. [16]	2016	Thyroidectomies performed by residents are not significantly longer and reveal no differences in length of stay or complication rates.
Gurrado et al. [17]	2016	Thyroidectomy can be safely performed by residents correctly supervised. Innovative gradual training in dedicated high-volume hospitals should be proposed in order to allow adequate autonomy for the residents and safeguard patient outcome.
Feeney et al. [18]	2017	A total of 84,711 cases were identified of which 45% involved trainee participation. There was not an increased overall or neurologic complication odds when a surgical trainee was involved.
Kshirsagar et al. [19]	2017	Resident participation in thyroid surgery was not associated with an increased 30-day postoperative complication rate.
Folsom et al. [20]	2017	Resident participation in hemithyroidectomy may be associated with increased operative duration, higher incidence of wound complications, and readmission.
Feeney et al. [18]	2017	A total of 84,711 thyroid and parathyroid surgical procedures cases were gathered from the American College of Surgeons National Surgical Quality Improvement Project database: of them, 45% involved trainee participation. No difference in the odds of overall patient complications or neurologic complications was observed when a trainee was involved. Mean operative time was found to be significantly different between attending only and junior and senior trainees. There was no significant difference in operative time between fellows and attending only.
Phitayakorn et al. [3]	2017	A survey was conducted among members of the AAES. A total of 92% of the respondents operate with residents. On average, they believed that the steps of a total thyroidectomy for benign disease and a well-localized parathyroidectomy could be performed by a postgraduate year 4 surgery resident. Specific steps that they thought might require more training included decisions to divide the strap muscles or leaving a drain. Approximately 66% of respondents thought that a postgraduate year 5 surgery resident could independently perform a total thyroidectomy for benign disease, but only 45% felt similarly for malignant thyroid disease; 79% thought that a postgraduate year 5 surgery resident could independently perform a parathyroidectomy.
Kay et al. [21]	2018	The number of endocrine surgeries performed by US otolaryngology residents has steadily grown from 1029 in 1996 to 1945 in 2015. The most significant growth occurred in endocrine surgery, in which there was a 288% increase from 18.4 surgeries per resident in 1996 to 71.5 surgeries per resident in 2015. The mean number of thyroidectomy surgeries performed by graduating residents increased from 16.5 in 1996 to 55.2 in 2015 (235 % increase), and parathyroid surgeries increased from 2.0 in 1996 to 16.3 in 2015 (715% increase).

The literature on the training of neck dissection is sparse. According to one report, surgical residents perform on average less than 2 neck dissections. Fellows in endocrine surgery perform more neck dissections but the numbers of such procedures vary between about 6 and 15 [9, 13]. According to one report, 12 neck dissections are considered appropriate to achieve the required competence [13].

### Training in parathyroid gland surgery

#### Survey results

Only a minority of the European surgical residency programs include parathyroid procedures during the training of graduating residents. The numbers of procedures assisted/performed varied widely ranging from 0 to 40 for assisted focused parathyroidectomy (FP) (median 0), from 0 to 30 for performed FP (median 0), from 0 to 10 for assisted bilateral neck exploration (BNE) (median 0), and from 0 to 5 for performed BNE (median 0). This was in contrast with the personal views of the respondents. Most of them considered that graduating surgeons should assist and perform a higher minimum number of FP (19 out of 24 (79.2%) and 17 out of 24 (70.8%), respectively) and BNE (18 out of 24 (75.0%) and 15 out of 24 (62.5%), respectively). In the opinion of the respondents, the minimum number of parathyroid procedures that should be assisted and performed during residency varied widely ranging from 0 to 40 for assisted FP (median 10), from 0 to 30 for performed FP (median 10), from 0 to 30 for assisted BNE (median 7.5), and from 0 to 30 for performed BNE (median 5).

For the available fellowship programs, national delegates reported a wide range of experience in parathyroid surgery. The number of procedures assisted/performed varied widely ranging from 3 to 100 for assisted FP (median 7.5), from 10 to 50 for performed FP (median 20), and from 5 to 20 for assisted BNE (median 10), and were 10 for performed BNE (median 10). All the responding national delegates from countries in which there are no endocrine surgery fellowships programs available agreed that a post-residency training is needed to achieve the expected competency.

In the opinion of the respondents, the minimum number of parathyroid procedures that should be assisted and performed during an endocrine surgery fellowship varied widely ranging from 5 to 100 for assisted FP (median 20), from 10 to 60 for performed FP (median 25), from 10 to 30 for assisted BNE (median 20), and from 10 to 30 for performed BNE (median 15).

#### Literature regarding training in parathyroid surgery

Using the search terms “parathyroidectomy AND training,” “parathyroidectomy AND residency,” and “parathyroidectomy AND fellowship,” 205 publications were identified on PubMed. However, only 12 of them were found to be relevant for this analysis [3, 5–7, 9–14, 18, 21]. The principal findings of the papers are summarized in Table 3.

All the studies included in the analysis were evaluating surgical training of US general surgery/otolaryngology residency programs or US endocrine surgery fellowship programs. No English literature was found on the training of general surgery/otolaryngology residents with regard to parathyroid surgery outside the USA.

In the USA, the data of the Residency Review Committee (RRC) of the Accreditation Council for Graduate Medical Education (ACGME) showed that there was a gradual but significant increase of the average numbers of endocrine surgical procedures from 1986 to 2008, parathyroidectomies in particular, performed by graduating general surgery residents (mean 4.1 parathyroid procedures in 1986 versus 9.6 in 2015) [5–7, 9–11, 14] (Table 3). Data of ACGME accredited otolaryngology—head and neck—surgery residency programs over 20 years (1996–2015) showed that the numbers of parathyroidectomies performed by graduating residents have steadily grown from 1.8 in 1996 to 16.3 in 2015 (an 800% increase) [3, 10, 14]. Zarebczan B. et al. [14] reported that most general surgery residents performed parathyroidectomies (and thyroidectomies) early during their training. Conversely, otolaryngology residents are performing most parathyroidectomies (and thyroidectomies) as chief residents (Table 3).

A recent survey conducted among AAES members reported that the respondents on average believe that all the steps of parathyroidectomy for a well-localized adenoma could be performed by a postgraduate 4-year surgery resident under direct supervision by an attending surgeon [3]. In addition, most of the respondents (79%) reported that a postgraduate final-year resident from their institution could autonomously perform a parathyroidectomy in a patient with concordant imaging [3] (Table 3).

In the late 1900s, in residency programs with one or more endocrine surgeon(s) in the teaching faculty, the number of parathyroidectomies performed by graduating general surgery residents was significantly higher as opposed to residency programs where such competency was missing [6] (Table 3). In addition, it has been reported that graduated general surgeons who choose to continue training in endocrine surgery performed significantly more endocrine cases during residency than the average graduating surgical resident (26 versus 9) [13] (Table 3).

Data on fellows training are less accurate since even in the USA there is no database for recognized fellowships like that of RRC [11]. Fellowship programs provide self-reported estimates on their operative volumes in thyroid, parathyroid, adrenal, neuroendocrine pancreas, and gastrointestinal endocrine procedures [11]. US fellowship programs showed significant differences in the numbers of endocrine operative cases performed at each program ranging from 10 to 500 parathyroidectomies [11]. During the early 2000s, the AAES recognized that endocrine surgery fellows graduated with a median of 80–90 parathyroid procedures (range 35–200) [9, 13] (Table 3). These numbers exceed the estimated numbers of performed cases to be competent (50, range 10–100) [13]. Given the rapid changes in parathyroid surgery over the past decade, in terms of preoperative localization studies, intraoperative PTH monitoring, and focused approaches in localized disease, AAES fellowship programs teach congruent management strategies that include focused dissection for localized disease and four-glands exploration if multiglandular disease is suspected, with intraoperative parathyroid hormone use and the intent to cure patients at the first surgery [22].

Trainees are involved in 45–73% of endocrine surgical operations performed at hospitals participating in the National Surgical Quality Improvement Program (NSQIP) of the American College of Surgeons [12, 18]. Between 2005 and 2008, senior residents assisted 36.7%, junior residents 32.9%, and endocrine surgery fellows 8.2% of 9101 parathyroidectomies [12]. Trainee-assisted operations were usually associated with longer operative time [12], especially if assisted by junior or senior residents [12, 18]. No difference in complications rate was observed when a trainee was involved [12, 18] (Table 3).

### Training in adrenal gland surgery

#### Survey results

There was a poor reply rate to the questions related to adrenal surgery. Only three respondents (from Poland, Slovenia, and Turkey) described their personal experience. Median (range) numbers were 5 (3–30) for open adrenalectomies performed, 5 (5–50) for open adrenalectomies assisted, 5 (2–30) for minimally invasive adrenalectomies performed, and 10 (5–50) for minimally invasive adrenalectomies assisted.

Nine respondents declared their personal views suggesting that during training one should be exposed to a median of 10 (2–30) open adrenalectomies performed and 10 (10–50) assisted and 10 (5–30) minimally invasive adrenalectomies performed and 10 (10–50) assisted.

The national requirements for becoming a fellow in endocrine surgery varied widely with a median of 10 (5–60) for performed adrenalectomies and 15 (10–100) for assisted adrenalectomies. The national delegates view was that a median of 20 performed and 20 assisted adrenalectomies should be required but once again their views varied widely (range 7–60 and 15–100, respectively). These figures are well over the workload of many units and reinforce the need to centralize training in adrenal surgery in a small number of units with workload of probably over 20 cases/year.

#### Literature regarding training in adrenal surgery

Data published on the current exposure of surgical residents to adrenal surgery is focused mainly on the North American situation but anecdotal evidence from most European countries is very similar. In a survey of 22 graduates from a general surgery residency program, over 50% did not feel competent performing several operations including adrenalectomy and this was an area considered to be in need of educational improvement at a program level (in addition to other operations such as abdominoperineal resection, transanal excision of tumors, transhiatal esophagectomy, and Whipple operations). Surprisingly, the analysis of their logbooks showed that increased case volume correlated with competence for adrenalectomy at very small numbers (3 vs. 1) though no established surgeon would accept that having been involved or having performed three adrenalectomies equates to competence of the procedure [23].

The exposure to small numbers of adrenal procedures has been reported repeatedly over the last decades. A study that examined the operative experience of general surgery residents in endocrine surgery for the academic years 1986–1987 to 1993–1994 found this training to be inadequate due to low operative volume. In a review of more recent data from the Resident Statistic Summaries from 1994–1995 to 2003–2004, the average number of adrenalectomies per resident was 1.46 (for endocrine pancreas, the average was 0.14). The most common number of any of these procedures performed by US graduates was zero [11]. A more recent analysis of case-log data from 2004 to 2009 for American urology residents showed no improvement while for Canadian residents there was a slightly improved exposure [24]

Exposure to endocrine (adrenal) surgery and mentorship during residency programs are powerful factors that influence residents to pursue careers in endocrine surgery. In a recent survey, fellows performed significantly more endocrine surgery cases during residency than the average graduating chief resident and mentorship was a critical factor in fellows’ decisions to pursue endocrine surgery. Fellows graduated with a median (range) of 13 (0–60) laparoscopic adrenal operations (compared with 150 (50–300) thyroid operations) [13]. When questioned about the “ideal” exposure they would aim for about 30. Fellows in minimally invasive surgery (MIS) listed the case volume for adrenalectomy as insufficient (similar to the situation for laparoscopic gastric banding, colectomy, common bile duct exploration, gastrectomy, esophagectomy, splenectomy, hepatectomy, nephrectomy, and pancreatectomy) [25].

It is abundantly clear that adrenalectomy is clearly established on the list of topics for which training remains poor in consecutive generations of trainees.

##### Impact of involving trainees in adrenal surgery

In a retrospective study using the American College of Surgeons National Surgical Quality Improvement Program database of 3219 adrenalectomies (735 open adrenalectomies and 2484 laparoscopic adrenalectomies), residents were involved in 2582 surgeries and senior residents or fellows performed the majority of the cases (85%). Resident participation led to a longer mean operative time but was not associated with significant differences in the operative outcomes of 30-day mortality or postoperative complications [26]. In contrast with these nationwide results, the operative time was reported to be similar when cases were performed under supervision of a dedicated trainer. In one study, 34% of the patients were operated by residents and 66% of the patients by a certified senior surgeon. There were no differences in operation time, intraoperative complications, postoperative complications, and length of stay between the patients operated by senior residents and those by certified surgeons [27].

The benefits of involving trainees in such cases were confirmed in a cohort study of 3694 patients who underwent adrenalectomy identified from the American College of Surgeons NSQIP database. A total of 732 adrenalectomies (20%) were performed by an attending surgeon with no trainee, 2315 (63%) involved a resident, and 647 (17%) involved a fellow. The participation of fellows was associated with fewer serious complications (7.9% with no trainee, 6.0% with residents, and 2.8% with fellows; *p* < 0.001). The odds of serious 30-day morbidity were lower when attending surgeons operated with residents (odds ratio = 0.63; 95% CI, 0.45–0.89). Fellow participation was associated with significantly lower odds of overall (odds ratio = 0.51; 95% CI, 0.32–0.82) and serious (odds ratio = 0.31; 95% CI, 0.17–0.57) morbidity. There was no significant association between trainee participation and 30-day mortality [28]. The same database was interrogated in another study of adrenal operations performed during 2005–2008 and no significant difference was found concerning the rates of wound infections, medical complications, reoperations, or overall morbidity between cases operated by the attending alone and those with the involvement of fellows/residents [12].

In summary, centers with adequate involvement in adrenal surgery should consider trainees as an integral and beneficial part of the surgical team and patients should feel and be reassured about their outcomes not being compromised.

##### Evidence of a learning curve for new techniques

After the report of laparoscopic adrenalectomy (LA) by Michel Gagner in early 1990s, this operation became the procedure of choice in the surgical management of most adrenal tumors. The “pioneers” never reported/analyzed their personal journey but later several groups aimed to define the learning curve for LA.

In one analysis, the first 100 LA procedures performed were divided into three, equal consecutive groups (*n* = 33, 33, and 34). The frequency of intraoperative complications in the intermediate and late group was significantly less compared with that in the early group (2 out of 33, 2 out of 34, and 7 out of 33, respectively; (*p* < 0.05). Similarly, the mean operating time was significantly reduced between the early (169 min) and both intermediate (116 min) and late (127 min) group (*p* < 0.005). The conversion rate was reduced between the three groups (3/33, 2/33, and 0/34, *p* = 0.06). According to this study, it seems that approximately 30 cases by an experienced laparoscopic surgeon are required to master the procedure [29].

Similar figures were quoted in a study from Italy who found that the operative time and conversion rates flattened their curves roughly at 30 and 40 procedures for right and left LA, respectively [30].

If a surgeon needs 30 cases (or more) before completing a personal learning curve and considering that the vast majority of surgeons performing LA undertake less than 6 cases/year, it would mean that more than 5 years would be needed for this surgeon to pass this learning curve. As such, it is even more concerning to learn that, in the UK, 186 of 222 surgeons involved in adrenal surgery perform less than 6 cases per year with a median of 1 case/year [31]. For this huge cohort of surgeons, their personal learning curve might not be completed during their entire career.

In the last decade, the wider adoption of retroperitoneoscopic adrenalectomy has offered the opportunity to analyze a learning curve for this new technique in centers already familiar with laparoscopic adrenalectomy. Several case series reported the experience of individual surgeons or centers in rather large number of patients (Table 4).

**Table 4:** Data published demonstrating the learning curve for retroperitoneoscopic adrenalectomy (RPA)

Reference	Total number of RPA cases	Changes in operating times
van Uitert et al. [32]	113	A median of 100 min in the first 20 patients decreased to 60 min after 40 patients, *p* < 0.05.
Cabalag et al. [33]	50	Operation time was decreased after 15 cases from 70.5 (54–85) min to median operative time 61 min.
Fukumoto et al. [34]	103	The learning curve stabilized at 30 cases. The cases were divided into two groups, the learning stage (LS) (cases 1–29) and master stage (MS) (cases 30–103) groups. In the LS group, the mean pneumoperitoneum time was 92 ± 35 min, which was significantly longer than the equivalent value for the MS group (55 ± 18 min, *p* < 0.001). In the LS group, the tumor size (≥50 mm) and the visceral fat area (VFA)/total fat area (TFA) ratio (≥ 0.49) were significantly associated with a prolonged pneumoperitoneum time (*p* = 0.046 and 0.046, respectively) (odds ratio 20.83 and 20.83, respectively). On the other hand, none of these factors were found to be associated with a prolonged pneumoperitoneum time in the MS group.
Barczyński et al. [35]	100	The steep segment of the learning curve took about 20–25 cases both during the invention phase of the RPA method and implementation phase in a different hospital 10 years later. Operations for pheochromocytoma, adrenal tumors larger than 3 cm in diameter, and male gender were found to affect the operating time in univariate analysis (mean 18.7 ± 5.4 vs 16.5 ± 4.6 vs 10.7 ± 3.2 min, respectively), whereas BMI was not a factor in this respect.

The learning curve is not easy to define based on a set number as local and personal/individual factors might induce significant variability. For example, in a report from four surgical teams from university centers in three different countries who analyzed their first 181 consecutive posterior retroperitoneoscopic adrenalectomies, competency was achieved after a range of 24–42 procedures [36].

Some expect that robotic adrenalectomy (RA) is going to be increasingly adopted in the coming years but currently few centers have embraced this technique as the associated costs do not offset any immediate clinical benefits. There is therefore very limited data to comment on a learning curve for RA. One paper comments on a significant reduction in operative times with gaining experience during exposure to 30 consecutive robot-assisted unilateral transperitoneal adrenalectomy procedures [37].

##### New/experimental training methods for adrenal surgery

It is expected that without formal structure, most units will use a similar model of staged clinical laparoscopic training program (without laboratory trainings) for beginners to perform LA. One paper reported the experience of five beginners with no previous experience in adrenalectomy who were randomly selected to receive the staged clinical laparoscopic training, including open retroperitoneal adrenalectomy or radical nephrectomy and mentor-initiated clinical laparoscopic training. The clinical data of the 15 LAs performed by each the trainees were collected and compared with the data from the initial 15 LAs of the mentor. All LAs were completed successfully, and no procedure required conversion to open surgery. The median operative time of the trainees was obviously shorter than the mentors’ time. The learning curve of the trainees was shorter compared with that of the mentor. The perioperative complication rate was similar between trainees and mentor. Beginners without laboratory trainings could perform LA safely and effectively after they participated in staged clinical laparoscopic training [38].

Staged laparoscopic training, including box-trainer, animal model, and mentor-initiated clinical training, was assessed using 5 beginners (postgraduate years 1–5) without previous experience in open adrenalectomy. During the clinical training, the trainees acted as the camera holder first and then selectively performed simple operations or parts. Finally, each of them performed 30 LAs independently under the mentor’s supervision using the technique of anatomic retroperitoneoscopic adrenalectomy. The learning curve among the trainees was shorter compared with that of the mentor. The authors concluded that it was safe and feasible for beginners without previous open counterpart experience to perform LA using staged training [39].

A surgeon-authored virtual reality (VR) training module authored by surgeons using the Toolkit for Illustration of Procedures in Surgery (TIPS) has been reported. A specialist surgeon authored the module, including force-feedback interactive simulation, and designed a quiz to test the knowledge of the key procedural steps. Five practicing surgeons, with 15 to 24 years of experience, peer reviewed and tested the module. In all, 14 residents and 9 fellows trained with the module and answered the quiz, pre-use and post-use. Participants received an overview during surgical grand rounds session and a 20-min one-on-one tutorial followed by 30 min of instruction in addition to a force-feedback interactive simulation session. Additionally, in answering the questionnaires, the trainees reflected on their learning experience and their experience with the TIPS framework. Correct quiz response rates on procedural steps improved significantly post-use over pre-use. In the questionnaire, 96% of the respondents stated that the TIPS module prepares them well or very well for the adrenalectomy, and 87% indicated that the module successfully teaches the steps of the procedure. All participants indicated that they preferred the module compared with training using purely physical props, one-on-one teaching, medical atlases, and video recordings [40, 41].

Mentorship is used when established surgeons embark on learning a new operative technique. One study objectively evaluated the impact of mentorship on the performance of RA and also compared it with LA. After implementing the use of RA, a retrospective review of the operative experience of two high-volume endocrine surgeons was performed. Both surgeons participated in a hands-on RA mentorship. Clinical presentation and perioperative outcomes were compared. Subgroup analysis was used to compare RA pre- and post-mentorship with LA. Sixty-one LAs and 31 RAs were included in the analysis. The mean operative time was 115 for LA versus 90 min for RA (*p* = 0.002). Ten patients were treated by RA in the pre-mentorship era versus 21 in the post-mentorship era. The mean operative time for the pre-mentorship group was 118 min, which decreased to 77 min post-mentorship (*p* < 0.0001). LOS also decreased from 2.0 to 1.2 days (*p* = 0.04) in the post-mentorship era [42].

Telementoring is a video-conferencing tool which can deliver expert opinion to physicians and their patients in remote locations. We report our experience with the use of telementoring as a technique to instruct in the performance of posterior retroperitoneoscopic adrenalectomy (PRA). Two consecutive PRAs conducted at Yale New Haven Hospital, New Haven, Connecticut, with telementored guidance from MD Anderson Cancer Center, Houston, Texas, were presented in a recent publication. The PRAs were performed after careful preparation of appropriate issues regarding cross-institutional telementoring. The procedures were performed quickly and safely. Loss of transmission occurred once but was re-established within seconds and was not disruptive to the surgical procedure. Patients were discharged within 48 h and without complications. In our experience, telementoring was convenient and effective in helping with the execution of a new surgical technique [43].

The same technique of telementoring was used to introduce PRA to Melbourne, Australia, where no highly experienced surgeon-mentors were available. A surgeon with experience of 12 PRA procedures attended from interstate, along with live telementoring via Skype video link by an overseas surgeon who had performed more than 200 PRA procedures, to mentor the surgeon-learner performing her first three cases. The operating surgeon’s first three PRA procedures proceeded uneventfully, with no complications, relatively short operative times, and one-night hospital stays for all three patients. It is important that the surgeon-learner has the skills and experience to complete the procedure using alternative techniques in the case of complications or technical failure [44].

Similarly, an Italian group described their experience with laparoscopic telementored adrenalectomy. Eight laparoscopic telementored adrenalectomies were performed between two separate operating sites 430 km apart. Six of these procedures were unilateral laparoscopic adrenalectomies, and one was bilateral. All cases were performed by an expert open surgeon who was skilled in laparoscopic procedure but who had no experience in laparoscopic adrenalectomy. All the procedures were successfully performed in a telementored fashion. The mean operative times, blood loss, and postoperative morbidity results were comparable with those for standard laparoscopic adrenalectomies reported in the literature [45].

### Training in surgery on gastro-entero-pancreatic neuroendocrine tumors

#### Survey results

There is a big variety concerning the numbers of operations for gastric, intestinal, and pancreatic procedures in the European countries. When it comes to training of surgical residents, the majority of operations performed on these organs (namely the stomach, the intestine, and the pancreas) are not performed on patients with NETs. This is a big difference as compared with thyroid gland, parathyroid gland, and adrenal gland procedures.

Our survey revealed that surgical residents according to the national surgical boards are required to perform between 0 and 15 gastric operations. However, the median number of required performed procedures was zero. Most national delegates felt that 4 (median) gastric operations should be performed by residents themselves. When asked for assisted gastric operations, the number was only slightly higher. According to the national delegates, 5 (median) operations were considered sufficient.

With regard to intestinal (small and large bowel) procedures, the numbers for required operations according to the national surgical boards were somewhat higher. In some countries, the minimum number of performed procedures was as high as 50. On average, however, only 5 (median) performed operations were required. The national delegates considered 12.5 (median) performed operations sufficient. Residents were required to assist 9 (median) intestinal operations while 10 (median) operations were considered appropriate by the national delegates.

In contrast to these numbers, pancreatic procedures are rather very rare. While the numbers for required performed (median 0) and assisted (median 0) operations were very low, slightly higher numbers (performed *n* = 2, assisted *n* = 5) were considered appropriate by the national delegates.

Adding the above numbers of gastric, intestinal, and pancreatic procedures, surgical residents should perform at least about 15 and assist another 15 GEP-NET operations according to the national delegates. The current number of required operations by the DES (performed *n* = 2, assisted *n* = 5) therefore appears very low.

As expected, the numbers considered to be appropriate for endocrine fellows were generally higher. While up to 30 performed and an additional 30 assisted gastric procedures were considered appropriate, the median number of appropriate procedures was 5 for performed and 8 for assisted operations. With regard to intestinal (small and large bowel) procedures, these numbers were slightly higher. The median numbers considered appropriate were 7 for performed and 10 for assisted operations. The median numbers were even relatively high for pancreatic procedures with 5 for performed and 20 for assisted procedures.

While the median numbers of operations considered to be appropriate overall were relatively high, the numbers that are required according to the national surgical boards were quite low in some countries. In addition, surgery of GEP-NETs is not part of a fellowship program in most European countries.

#### Literature regarding training in GEP-NET

GEP-NETs are rather rare entities and their surgical treatment is not universally managed. Gastric NET may be surgically treated by general surgeons, upper-gastrointestinal surgeons, and endocrine surgeons. Enteric NETs may be operated on by general surgeons, lower gastrointestinal surgeons, colorectal surgeons, and endocrine surgeons. Pancreatic NETs may be in the same hands as gastric NETs. Since the surgical treatment of these tumors is distributed among various surgical subspecialties, any general statement is difficult to make. It has been shown that defining the right surgical fellowship program can be a challenge in itself [46].

Literature addressing training of surgical residents or fellows with regard to gastro-entero-pancreatic neuroendocrine tumors is almost non-existing. This is somewhat surprising owing to the fact that the surgical strategy on neuroendocrine tumors may differ somewhat from that used in their more common adenocarcinoma counterparts. This applies both to the resection of primary tumors and also to metastases [47]. Thus, there would be a lot to teach. Nevertheless, some literature exists on the training of both residents and fellows with regard to gastric, colorectal, and hepato-pancreato-biliary surgery in general and this will be addressed below. Of course, the procedures analyzed may differ a lot from those used in patients with GEP-NETs but the findings may give some important clues.

##### Gastric operations

With regard to training of gastric operations, many studies address bariatric procedures. It has been shown that a stepwise approach consisting of (1) the creation of the gastric pouch, (2) identification of the ligament of Treitz, measuring the biliopancreatic limb and creating the stapled gastrojejunostomy, (3) laparoscopic suture closure of the linear stapled gastrojejunal anastomosis, (4) measuring the alimentary limb and creating the stapled jejunojejunostomy, and (5) laparoscopic suture closure of the linear stapled jejunojejunal anastomosis can teach residents to perform a laparoscopic Roux-en-Y gastric bypass in an efficient and safe way [48]. This was also shown for robotic laparoscopic gastric banding surgery [49]. Animal models have been successfully used to train 1-year residents performing a gastrojejunostomy [50] and for some gastric procedures (e.g., laparoscopic pyloromyotomy), 3D models have been shown to be of help to teach trainees [51].

Some investigators reported that resident participation seems to lead to an increase of the incidence of superficial site infection [52, 53] most likely due to longer operating times but this was considered to be clinically insignificant.

When it comes to laparoscopic gastric cancer surgery, it has been stated that the current exposure of trainees might be considered insufficient [54].

##### Intestinal operations

A study from Switzerland showed that less than 5% of all segmental colectomies between 2006 and 2015 were performed by residents [55]. During the same time period, the annual number of graduates increased by more than 100%. It is therefore not surprising that a low rate of competence and subsequently confidence was found in another study analyzing laparoscopic colorectal surgery [56]. Earlier exposure of surgical residents has been demanded with regard to laparoscopic colorectal surgery [57]. Possible solutions could include video-trainers and animal models [56].

With regard to laparoscopic colorectal surgery, it has been shown that residents during their final years very well can achieve results that do not affect patient safety and short-term outcome adversely [58]. And some authors did not find any difference in intraoperative adverse effects during colonic procedures when comparing consultants with trainees [59].

Obviously, fellows are more likely to be confronted with the required number of operations. A study analyzing independency of fellows performing laparoscopic colorectal procedures showed that it can be achieved but quite a number of procedures (more than 50) may be necessary [60, 61]. Still, it has been shown that surgeons having reached such a competence immediately thereafter can successfully train other colleagues [62]. One challenge is that fellowship programs compete with residence programs. In one study, only a few program directors considered fellow programs having a positive effect on the residence program [63].

##### Pancreatic operations

In 1996, Harness and colleagues published a study from the USA on the experience of residents with regard to some rare endocrine diseases including endocrine pancreatic surgery [64]. Over a timespan from 1986 to 1994, the maximum number of endocrine pancreatic procedures ranged from 3 to 10. However, almost 85% of the residents did not perform any such procedure at all. The authors concluded that most resident graduates have little or no experience with any of these procedures.

A survey sent to program directors of general surgery in the USA to determine how Accreditation Council for Graduate Medical Education (ACGME) hepato-pancreato-biliary (HPB) requirements were met revealed that about one-fourth of the directors were required to send their residents to other facilities in order to be able to offer the required number of operations [63]. While residents completed about 70% of all operations, less than 50% were considered competent by the program directors. This may not be surprising, since more than 50% of all operations by the residents were performed during the final year while less than 50% were performed during resident years 1–4 [65]. Residents performed less than 10 pancreas and liver operations giving them practically no chance to become independent and subsequently competent [65]. It would be desirable to expose residents sooner to more complex procedures [66]. For these more complex procedures, a stepwise approach can achieve satisfying results [67]. With regard to training, the usefulness of 3D modelling (pancreas) has been shown [68].

While residents used to complete only very few pancreatic procedures [69], current programs in the USA seem to be able to offer fellows quite sufficient numbers of operations with a median of close to 50 liver cases and more than 60 pancreatic cases [70].

Operations performed by fellows have been shown to be associated with a higher morbidity, in particular due to an increased frequency of infections, while the mortality rate did not differ significantly as compared with experiences surgeons [71, 72].

It has been shown that fellows and residents compete with each other but that residence training in laparoscopic surgery in the absence of fellows may not affect patients’ outcome [73]. This is another argument in favor with teaching as early as possible. When the number of cases is high enough, fellows do not negatively affect the training of surgical residents [74].

## Discussion

This study represents the first European survey evaluating the training in endocrine surgical procedures of general surgery/otolaryngology residents and endocrine surgery fellows. The work was undertaken on the background of a paucity of data regarding training in endocrine surgical procedures outside the USA.

The analysis of the existing literature confirms that the exposure of graduating residents to endocrine surgical procedures is far from what should be considered adequate to achieve individual competency [13] (Table 3) and what is recommended to be eligible for applying for the EBSQ of the DES (Table 1). This is true for thyroid and parathyroid procedures, but is particularly evident for less common and probably more demanding operations, such as GEP-NET resections and lateral neck dissection [11, 13, 64]. The only exception could be the US otolaryngology—head and neck—residency programs that in recent years have exposed graduating residents to increasing neck endocrine surgical activities, consistent with the minimum for achieving surgical competency [21]. In addition, non-European endocrine surgeons (USA, Canada, and South Africa) who responded to the present survey, depicted a minimal exposure of general surgery residents to endocrine surgical procedures (less than 5 procedures in most of the cases; data not included in the present analysis).

While it has been extensively demonstrated that involving residents in endocrine surgery procedures is associated with longer operative times, it is not associated with clinically significant increased complication rates [12, 17, 18]. In our opinion, slightly longer operating times are an affordable price to pay in order to obtain adequate resident training. Obviously, resident involvement should be progressive and standardized based on the complexity of the surgical steps and of the surgical procedures [3]. It has been shown that comprehensive surgical coaching comprised performance analysis, debriefing, feedback, and behavior modelling can enhance the results in skill acquisition as compared with conventional training [75].

In addition, the number of procedures performed and assisted represents only a rough tool to evaluate graduating residents’ proficiency. Tools to objectively evaluate surgical skill could be useful but they are yet to be validated in current practice [76].

Of note, residents who graduated in programs, in which one or more faculty members were endocrine surgeons, were significantly more exposed and trained in endocrine surgical procedures comparing with the average general surgery residents [6, 13]. As a consequence, one should argue that the numbers reported for endocrine surgical activity during residency from the present survey could be overestimated. Indeed, this is not a report based on official data of residents’ surgery case logs obtained from official institutions (which, to our knowledge, do not exist for most of the European countries) but based on self-estimated reports of the national delegates of the DES/ESES. By definition, all the respondents are active “endocrine surgeons” and could be influenced by their own experience. Obviously, this could be considered the main limitation of the present evaluation.

It is evident that general surgery specialists who apply for endocrine surgery fellowships during residency were more exposed and trained in endocrine surgical procedures [13]. This underlines the importance of mentorship and tutorship during the training of future surgeons. Exposure starts during medical school and medical students during their surgical rotations are probably underexposed to endocrine surgery, even those who will opt for surgical residency [77]. Since this paucity of exposure may have significant detrimental educational and career ramifications, it would be expected and suggested that endocrine surgeons, especially those who are involved in academic and tutoring activities, would be more active in exposing medical students and resident to endocrine surgery procedures. This is in line with the results of the current survey, since the vast majority, if not all, of the respondents expected and advocated an increase in the exposure of surgical residents to endocrine surgery procedures, both as assistant and as performing surgeon.

Considering the usually inadequate endocrine surgical training during residency, surgery residents wishing to practice endocrine surgery may benefit from additional training in their final year or dedicated fellowship training [14]. Many studies published in recent years showed a relationship between hospital/surgeon volume and patient outcomes which highlights the importance of advanced postgraduate training in endocrine surgery [78–83]. Two years of additional fellowship training in thyroid and endocrine surgery is now being advocated by an increasing number of national endocrine surgical associations as the best way to prepare surgeons for society’s needs for highly skilled, competent thyroid surgeons of the future [1]. Indeed, recognized endocrine surgery fellowships seem to provide the necessary additional surgical experience [9, 11, 13] and are usually able also to give competence on concepts and approaches, which are relevant in the modern endocrine surgical practice, including minimally invasive techniques and intraoperative adjuncts (i.e., IOPTH monitoring, IONM, etc.) [22, 84]. However, graduated fellows do not feel prepared and competent in all the surgical procedures they could be faced with in individual practice, especially regarding those procedures that are less common and, presumably, more complex, including pancreatic resection and neck dissection [13].

In addition, there is a wide discrepancy in different endocrine surgery fellowship programs, both at national and international levels. This is evident from the analysis of the pertinent literature (which, of note, considered only the endocrine surgery fellowship programs recognized by the IAES or the AAES) and from the present survey. Not all graduated endocrine surgery fellows are trained in the same way and with the same objectives and curricula.

Due to the paucity of recognized fellowship programs and to the heterogeneity of the existing ones, an implementation and standardization of fellowship programs could be advocated at national and/or international levels. It has been noted many years ago that the surgeon plays an important prognostic factor in endocrine surgical diseases [85]. Therefore, proper training of the “successors” should be in the main interest of every practicing surgeon involved in endocrine surgery. As much as one is responsible for the good outcome of the care of current patients, one is equally responsible for the training of those who will take care of future patients.

The survey clearly showed that most of the current numbers of performed and assisted operations required to be eligible to be examined by the DES are comparable with what European national surgical boards have defined and what European national delegates consider to be appropriate (Fig. 1a). However, some of the current numbers as defined by the DES are far below what both European national surgical boards and national delegates do recommend. The DES requires only 2 performed central and lateral lymph node dissections each. This is far beyond what national surgical boards and national delegates recommend. Between 10 and 15 performed central and lateral lymph node dissections appear to be more appropriate (Fig. 1e, g).

Established surgeons with a practice in adrenal surgery should have a minimum workload of 6 cases/year. When this cannot be secured, referral to alternative medical centers or centralization of practice should be compulsory. In view of a recognized learning curve for new techniques, retroperitoneoscopic adrenalectomy should be adopted in centers with a minimum of 12–20 cases/year. Training in robotic adrenal surgery should be provided in the context of a larger expertise in robotic surgery and in centers with annual workload of > 20 adrenal cases.

Based on national differences in service provision, patients with GEP-NETs should be cared by in centers with a minimum of 10–20 cases per year and trainees with a specific interest in this type of (rare) pathology work in such recognized centers.

However, it is important to underline that operating numbers often are used as an indicator for surgical experience of trainees despite the fact that it has been demonstrated that there is no evidence in any surgical specialty that thresholds of operative experience are equivalent to a particular level of ability [86]. Competence in a certain surgical subspecialty should be regarded as the combination of detailed basic and clinical knowledge and reasoning and clinical and surgical experience. Consequently, although the demonstration of operative experience would seem to be a reliable criterion for evaluating competence in endocrine surgery practice, probably more efficient methods of achieving and evaluating competence would be necessary.

Of note, EBSQ examination for endocrine (neck) surgery qualification evaluates not only surgical experience, based on operative log book of candidates, but also basic knowledge and clinical reasoning. As a summary of these findings, the authors have formulated a set of statements (Table 5) that were discussed during a plenary session of the ESES 2019 meeting.

**Table 5:** Statements regarding the training of fellows in (neck) endocrine surgery

(a) Training in endocrine surgery should be performed in units that perform a minimum of 100 thyroid, 50 parathyroid, 15 adrenal, and/or 10 GEP-NET operations yearly.	
(b) Fellowships of 1–2 years are recommended part of postgraduate training for those who intend to specialize in endocrine surgery.	
(c) Fellows should be expected to have been main operator in a minimum of 50 thyroid operation, 10 (central or lateral) lymph node dissections, 15 parathyroid, 5 adrenal, and 5 GEP-NET operations.	
(d) Fellows are encouraged to be examined on the national or European level.	
(e) The European Society of Endocrine Surgeons (ESES) will support trainees with a dedicated interest by providing the JF Henry Travelling Fellowship.	

## Conclusion

Surgical trainees with an interest in endocrine surgery should work on a unit with a recognized interest in this specialty so that appropriate clinical exposure can be secured. They should aim to obtain formal certification of training through their national examination and/or the DES examination.

Fellowships in endocrine surgery should be available only in large regional centers where multidisciplinary approach to complex endocrine cases would offer the trainees the best chance for learning a comprehensive management of endocrine surgical diseases.
